# Supplementary material for: Modeling glioblastoma heterogeneity as a dynamic network of cell states
Source: Mol Syst Biol. 2021 Sep 16;17(9):e10105. doi: 10.15252/msb.202010105 (PMC8444284; doi:10.15252/msb.202010105)
Supplement: Supplementary file 5 — Source Data for Figure 3 [file MSB-17-e10105-s001.zip › Figure3A_sourcedata/GSEA_3065/hallmarks_state3.GseaPreranked.1623416274744/HALLMARK_REACTIVE_OXYGEN_SPECIES_PATHWAY.html]

Details for gene set HALLMARK\_REACTIVE\_OXYGEN\_SPECIES\_PATHWAY[GSEA]

|  || Dataset | state3 |
| Phenotype | NoPhenotypeAvailable |
| Upregulated in class | na\_neg |
| GeneSet | HALLMARK\_REACTIVE\_OXYGEN\_SPECIES\_PATHWAY |
| Enrichment Score (ES) | -0.63220656 |
| Normalized Enrichment Score (NES) | -1.869918 |
| Nominal p-value | 0.0 |
| FDR q-value | 5.093138E-4 |
| FWER p-Value | 0.004 |
Table: GSEA Results Summary

  

Fig 1: Enrichment plot: HALLMARK\_REACTIVE\_OXYGEN\_SPECIES\_PATHWAY      
 Profile of the Running ES Score & Positions of GeneSet Members on the Rank Ordered List

  

| PROBE | GENE SYMBOL | GENE\_TITLE | RANK IN GENE LIST | RANK METRIC SCORE | RUNNING ES | CORE ENRICHMENT || 1 | PRDX4 |  |  | 41 | 0.455 | 0.0607 | No |
| 2 | SELENOS |  |  | 213 | 0.276 | 0.0808 | No |
| 3 | PRNP |  |  | 609 | 0.185 | 0.0621 | No |
| 4 | SCAF4 |  |  | 1005 | 0.138 | 0.0366 | No |
| 5 | ERCC2 |  |  | 3049 | 0.040 | -0.1920 | No |
| 6 | NDUFS2 |  |  | 3331 | 0.032 | -0.2196 | No |
| 7 | MSRA |  |  | 3910 | 0.017 | -0.2834 | No |
| 8 | GCLM |  |  | 4026 | 0.014 | -0.2945 | No |
| 9 | PFKP |  |  | 4040 | 0.014 | -0.2940 | No |
| 10 | JUNB |  |  | 4467 | 0.002 | -0.3426 | No |
| 11 | SBNO2 |  |  | 5024 | -0.012 | -0.4047 | No |
| 12 | STK25 |  |  | 5209 | -0.016 | -0.4235 | No |
| 13 | OXSR1 |  |  | 5310 | -0.019 | -0.4323 | No |
| 14 | PTPA |  |  | 5484 | -0.024 | -0.4487 | No |
| 15 | CAT |  |  | 5510 | -0.024 | -0.4481 | No |
| 16 | SOD2 |  |  | 5817 | -0.033 | -0.4784 | No |
| 17 | GSR |  |  | 5892 | -0.035 | -0.4818 | No |
| 18 | TXNRD1 |  |  | 6009 | -0.040 | -0.4894 | No |
| 19 | TXNRD2 |  |  | 6132 | -0.044 | -0.4971 | No |
| 20 | CDKN2D |  |  | 6491 | -0.056 | -0.5301 | No |
| 21 | PDLIM1 |  |  | 6965 | -0.079 | -0.5730 | No |
| 22 | HMOX2 |  |  | 7076 | -0.085 | -0.5733 | No |
| 23 | GLRX |  |  | 7283 | -0.099 | -0.5827 | No |
| 24 | GLRX2 |  |  | 7491 | -0.116 | -0.5898 | No |
| 25 | PRDX2 |  |  | 7855 | -0.159 | -0.6086 | Yes |
| 26 | G6PD |  |  | 7860 | -0.160 | -0.5861 | Yes |
| 27 | NQO1 |  |  | 8263 | -0.244 | -0.5972 | Yes |
| 28 | NDUFA6 |  |  | 8407 | -0.298 | -0.5707 | Yes |
| 29 | PRDX6 |  |  | 8410 | -0.300 | -0.5278 | Yes |
| 30 | SOD1 |  |  | 8526 | -0.349 | -0.4909 | Yes |
| 31 | NDUFB4 |  |  | 8560 | -0.372 | -0.4413 | Yes |
| 32 | LAMTOR5 |  |  | 8609 | -0.410 | -0.3878 | Yes |
| 33 | TXN |  |  | 8662 | -0.467 | -0.3266 | Yes |
| 34 | ATOX1 |  |  | 8705 | -0.526 | -0.2557 | Yes |
| 35 | GPX4 |  |  | 8742 | -0.690 | -0.1607 | Yes |
| 36 | FTL |  |  | 8751 | -1.124 | 0.0000 | Yes |
Table: GSEA details [plain text format]

  

Fig 2: HALLMARK\_REACTIVE\_OXYGEN\_SPECIES\_PATHWAY: Random ES distribution      
 Gene set null distribution of ES for **HALLMARK\_REACTIVE\_OXYGEN\_SPECIES\_PATHWAY**
